# Identification of key lncRNAs in age-related macular degeneration through integrated bioinformatics and experimental validation

**DOI:** 10.18632/aging.205656

**Published:** 2024-03-14

**Authors:** Yuying Ji, Chengguo Zuo, Nanying Liao, Liwei Yao, Ruijun Yang, Hui Chen, Feng Wen

**Affiliations:** 1 State Key Laboratory of Ophthalmology, Zhongshan Ophthalmic Center, Sun Yat-Sen University, Guangdong Provincial Key Laboratory of Ophthalmology and Visual Science, Guangdong Provincial Clinical Research Center for Ocular Diseases, Guangzhou 510060, China

**Keywords:** noncoding RNA, age-related macular degeneration, bioinformatics, LINC00276, senescence

## Abstract

This study aimed to identify key long noncoding RNAs (lncRNAs) in age-related macular degeneration (AMD) patients and to identify relevant pathological mechanisms of AMD development. We identified 407 differentially expressed mRNAs and 429 differentially expressed lncRNAs in retinal pigment epithelium (RPE) and retina in the macular region of AMD patients versus controls (P < 0.05 and |log2FC| > 0.585) from GSE135092. A total of 14 key differentially expressed mRNAs were obtained through external data validation from GSE115828. A miRNA-mRNA and miRNA-lncRNA network containing 52 lncRNA nodes, 49 miRNA nodes, 14 mRNA nodes and 351 edges was constructed via integrated analysis of these components. Finally, the LINC00276-miR-619-5p-IFIT3 axis was identified via protein-protein network analysis. In the t-BH-induced ARPE-19 senescent cell model, LINC00276 and IFIT3 were downregulated. Overexpression of LINC00276 could accelerate cell migration in combination with IFIT3 upregulation. This compelling finding suggests that LINC00276 plays an influential role in the progression of AMD, potentially through modulating senescence processes, thereby setting a foundation for future investigative efforts to verify this relationship.

## INTRODUCTION

The complicated neurodegenerative disease known as age-related macular degeneration (AMD) has both hereditary and environmental factors. AMD is the leading cause of irreversible visual loss [1]. The complement system, lipid metabolism, and extracellular matrix organization are three potential biological processes in the pathogenesis of AMD [2]. To anticipate which patients may develop this disease’s advanced manifestations, significant progress has also been made in identifying the genetic risk variants for this disease. Despite significant advancements in recent years, it is still difficult to pinpoint the root causes and underlying mechanisms of AMD.

**Figure 1:**
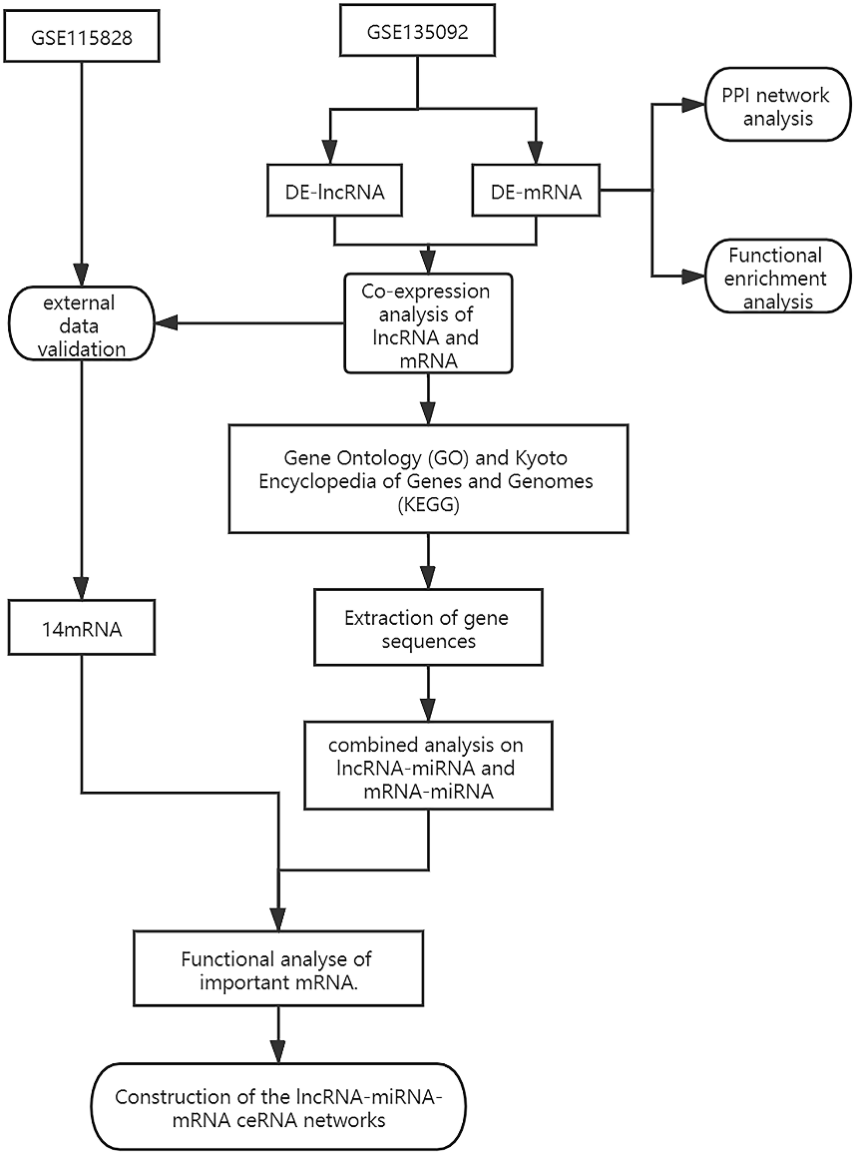
The diagram showing how the lncRNA-miRNA-mRNA network is built.

Long noncoding RNAs (lncRNAs) are a subclass of noncoding RNAs that include more than 200 nucleotides and are crucial for a variety of biological processes [3]. More than 200 lncRNAs are dysregulated in the RPE tissues of AMD patients [4], these include lncRNA HDAC4-AS1 [5] and lncRNA LINC00167 [6], which are involved in AMD development. Current research suggests that lncRNAs regulate cell migration and adherence in neovascularization through focal adhesion signaling pathways [7]. These investigations, however, merely skim the surface of lncRNAs’ potential roles in AMD etiology. Further clarification of the relationship between lncRNAs and pathogenesis might provide novel insight into the prevention and treatment of AMD. In the current study, a regulatory network of lncRNA-miRNA-mRNA was identified (Figure 1) in AMD patients and we attempted to identify relevant pathological mechanisms of AMD development. *In vitro*, we confirmed that LINC00276 enhances the migration of ARPE-19 cells by Transwell assay and scratch assay. Our findings may provide a novel therapeutic target for AMD.

## RESULTS

### Identification of differentially expressed lncRNAs and mRNAs

**Figure 2:**
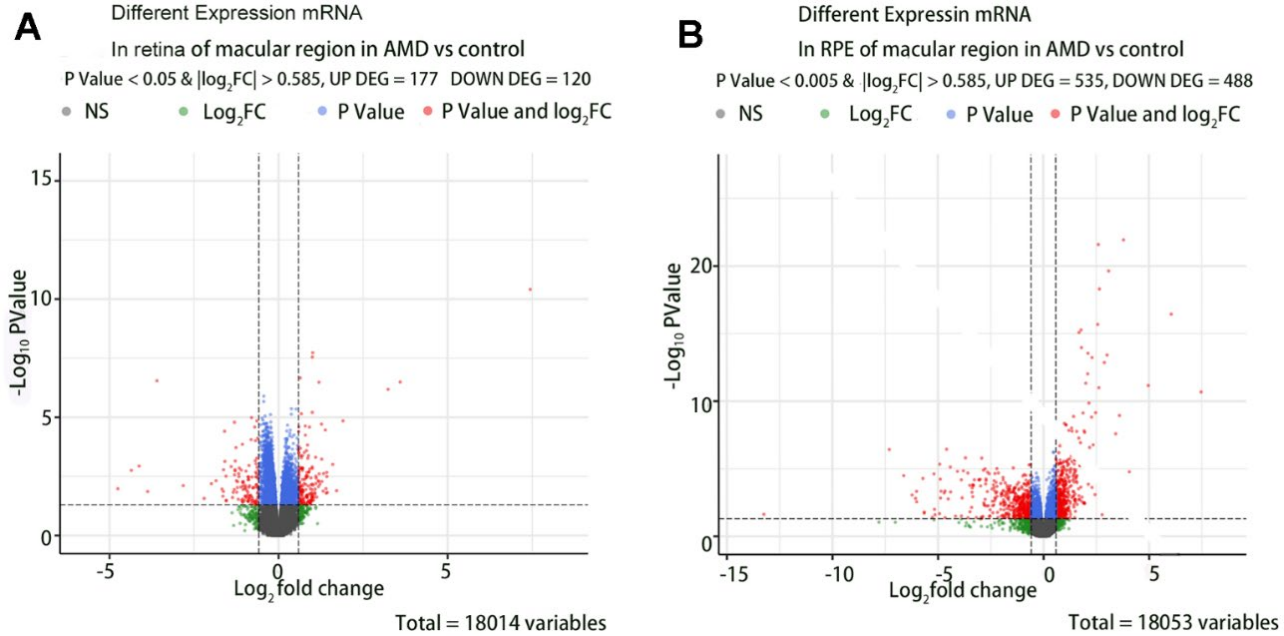
**The volcano map of the DE-mRNAs in retina and RPE in macular region of AMD vs controls.** (**A**) DE-mRNAs in retina of macular region in AMD vs controls. (**B**) DE-mRNAs in RPE of macular region in AMD vs controls.

A total of 407 DE-mRNAs and 429 differentially expressed lncRNAs were identified when comparing the retina and RPE in the macular region of AMD patients to those of controls (P < 0.05 and |log_2_FC| > 0.585). We visualized the differential gene analysis results with a volcano map plot. Figure 2 shows the volcano map of the DE-mRNAs in group (1) and group (2).

### Gene ontology (GO) enrichment and Kyoto Encyclopedia of Genes and Genomes (KEGG) pathway analysis of DE-mRNAs

**Figure 3:**
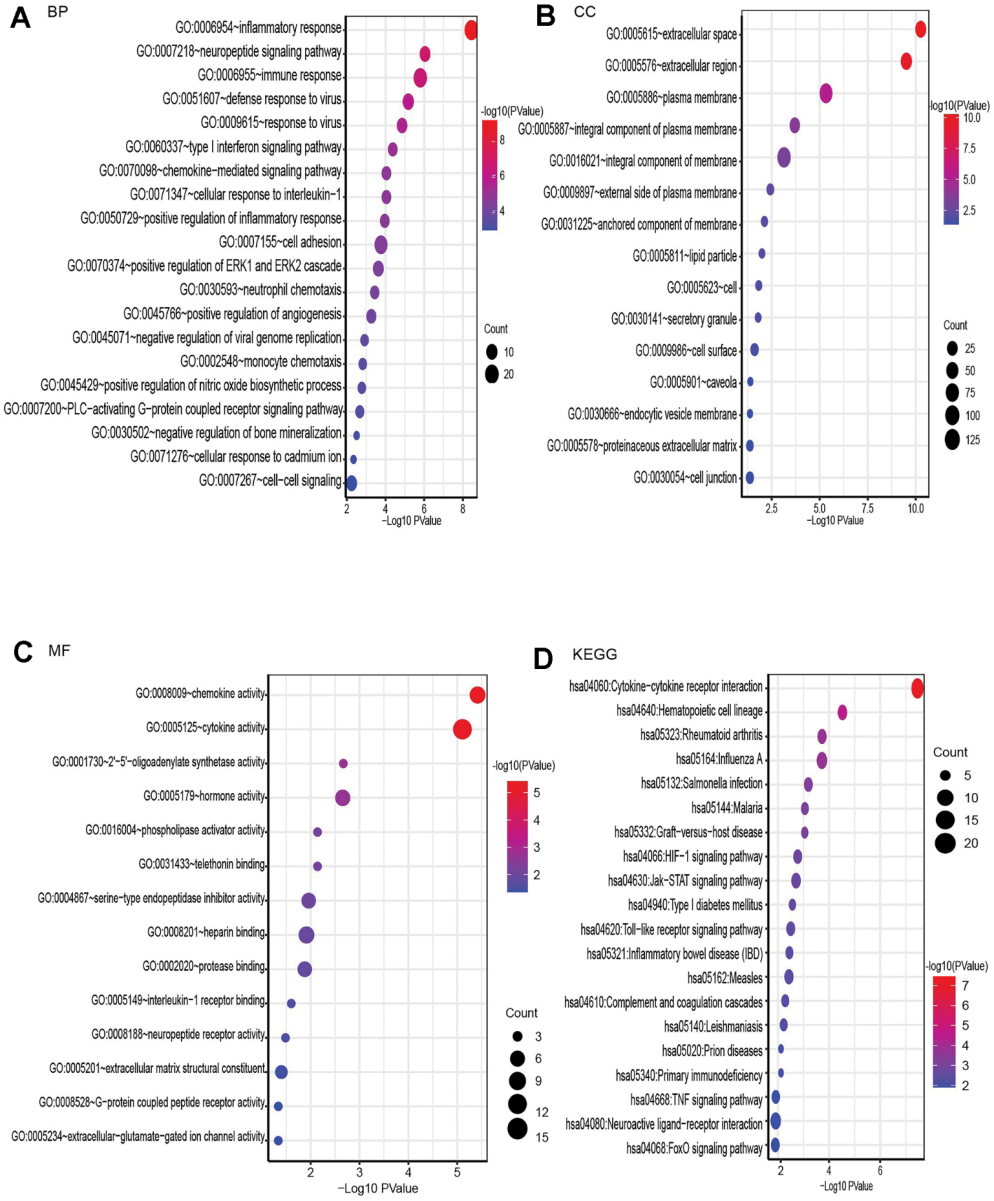
**GO enrichment and KEGG pathway analysis of differentially expressed mRNAs, BP for biological process, MF for molecular function, and CC for cellular component.** (**A**) Bubble plot of BP. (**B**) Bubble plot of CC. (**C**) Bubble plot of MF. (**D**) Bubble plot of KEGG.

Using Database for Annotation, Visualization, and Integrated Discovery (DAVID), GO and KEGG analyses were performed on these genes to investigate their activities and signaling networks. The top 20 GO and KEGG pathway enrichment terms were identified, as shown in Figure 3. The GO functions that were the most enriched were the inflammatory response (GO: 0006954), neuropeptide signaling pathway (GO: 0007218) and immune response (GO: 0006955), which involved 29, 13 and 26 differentially expressed genes (DEGs), respectively.

The top Kyoto Encyclopedia of Genes and Genomes (KEGG) signaling pathway was associated with cytokine-cytokine receptor interaction and involved 23 DEGs, as shown in the bubble chart in Figure 3.

### Protein-protein interaction (PPI) network construction

**Figure 4:**
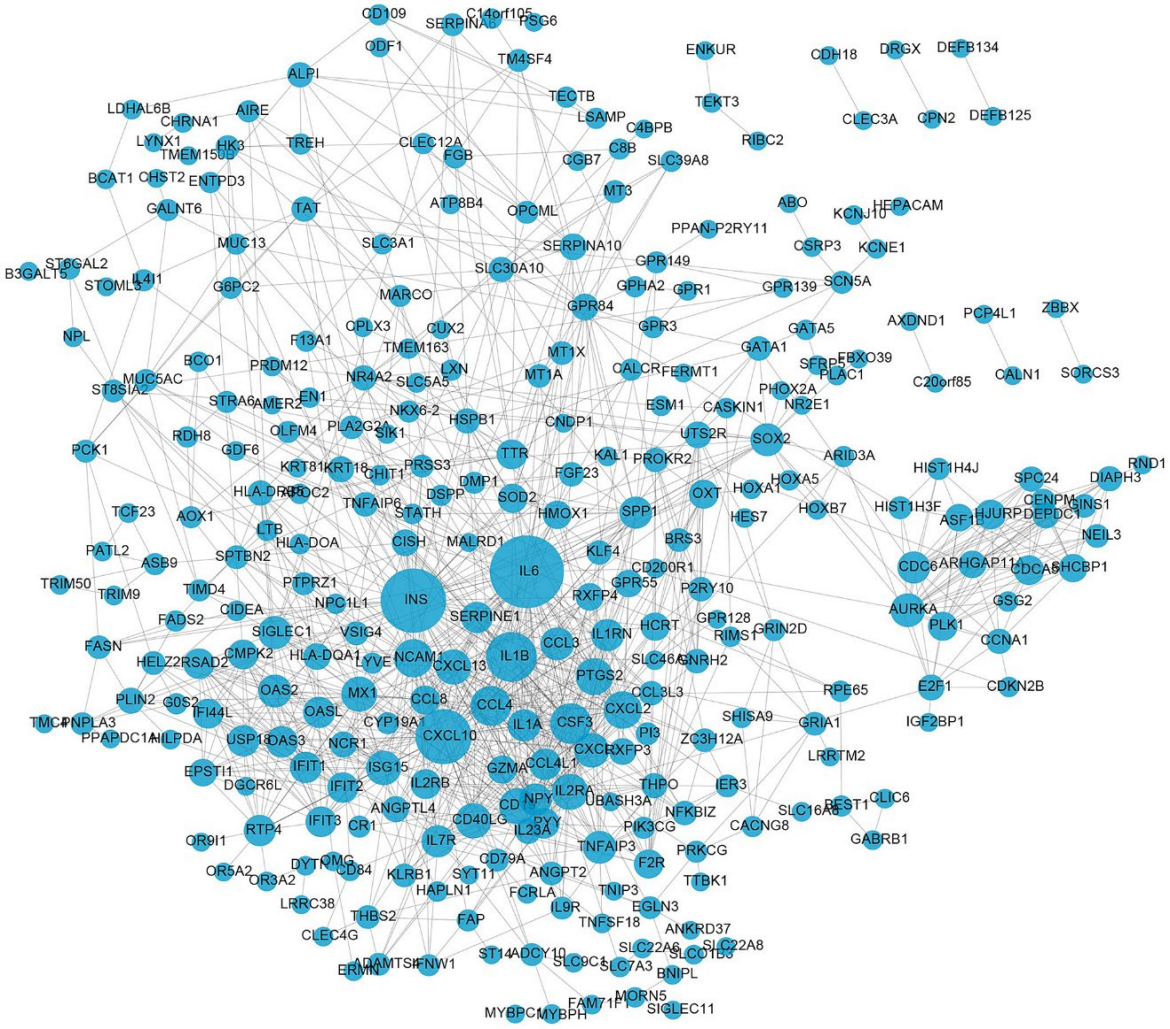
**PPI network construction of DE-mRNAs.** The circles represent differentially expressed mRNAs. The size of the circle signifies the extent of interactions (degree) a gene has with others. The larger the circle, the greater the number of interactions it has.

From the visualized results obtained by Cytoscape, as shown in Figure 4, we could see that in the PPI network, there were 296 nodes and 1072 edges. The genes in the figure that have more protein interactions with other nodes were mostly immune-related genes (such as IL6, IL1A, IL1B, ING, CXCL10, etc.), indicating that immune cells are crucial for the emergence of AMD. These results were consistent with the GO enrichment and KEGG pathway analysis results.

### Functional analysis of lncRNAs

**Figure 5:**
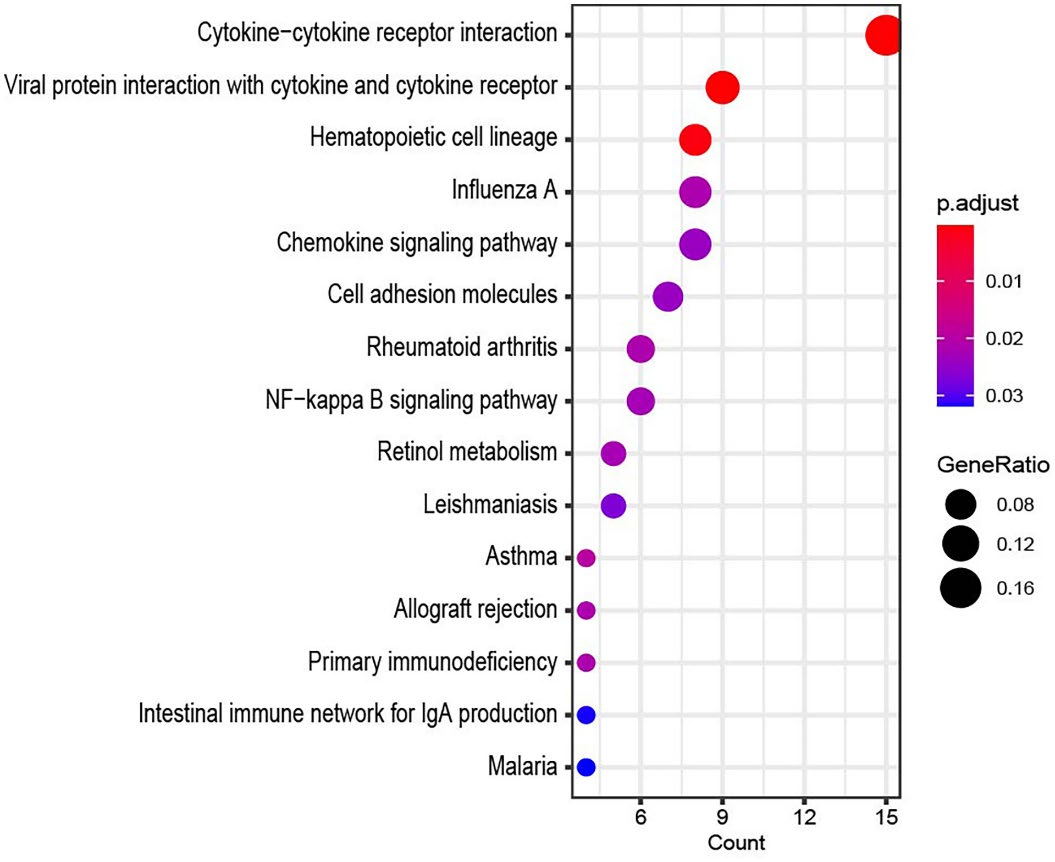
The KEGG result of LINC00276.

Based on the target gene information regulated by lncRNAs, GO enrichment and KEGG pathway analyses of each lncRNA were performed. Figure 5 shows the KEGG results for LINC00276. The top KEGG pathway of LINC00276 was cytokine-cytokine receptor interaction.

### External data validation and mRNA function analysis

**Figure 6:**
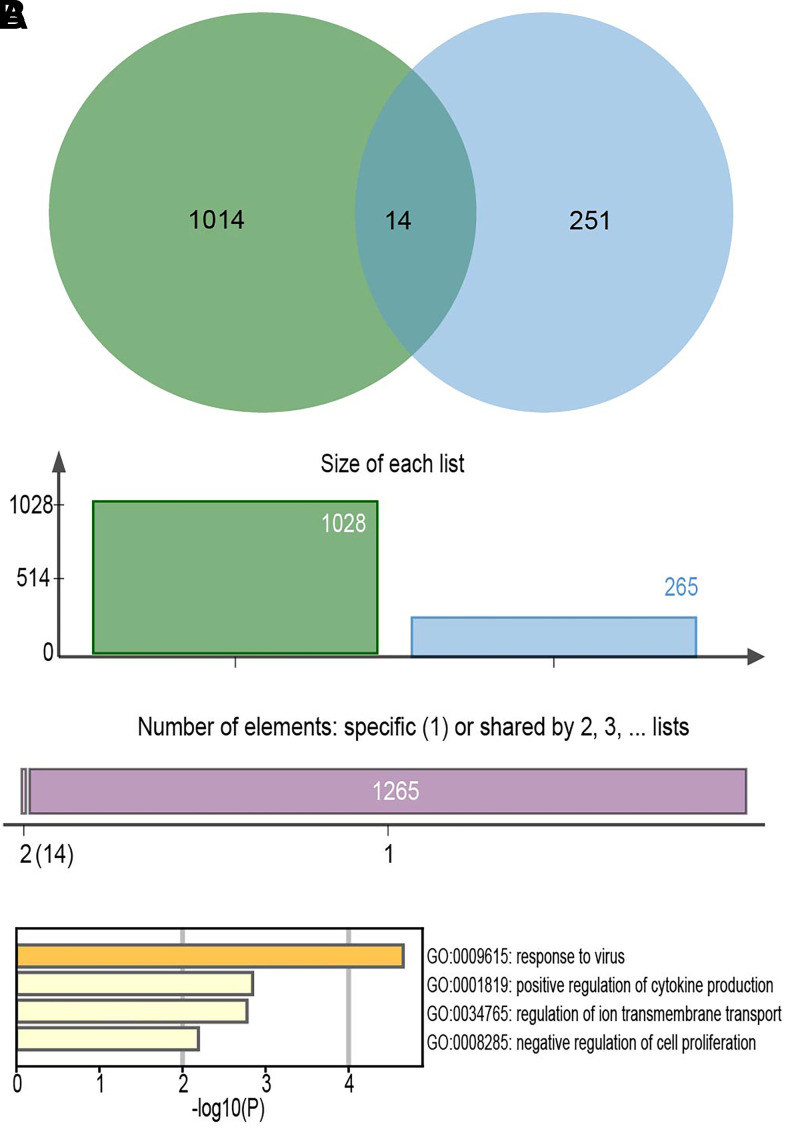
**The Venn diagrams of overlapped mRNAs and the function analysis.** (**A**) A total of 14 key DE-mRNAs were obtained from the intersection of the mRNAs co-expressed in GSE135092 and the differentially expressed mRNAs obtained from GSE115828. (**B**) The functional analyses of the 14 key DE-mRNAs.

From the intersection of the mRNAs co-expressed in GSE135092 and the differentially expressed mRNAs obtained from GSE115828, a total of 14 key DE- mRNAs were obtained (Figure 6A). They were PODN, F2R, SLC38A11, HSPB1, CLIC6, CISH, GDPD2, RSAD2, IFIT3, PCDHA4, IGSF1, KCNH5, IFI44L, and GDF6. They were most likely implicated in the action of the viral response and the positive regulation of cytokine production, according to functional analyses (Figure 6B).

### Construction of the lncRNA-miRNA-mRNA competing endogenous RNA (ceRNA) networks

**Figure 7:**
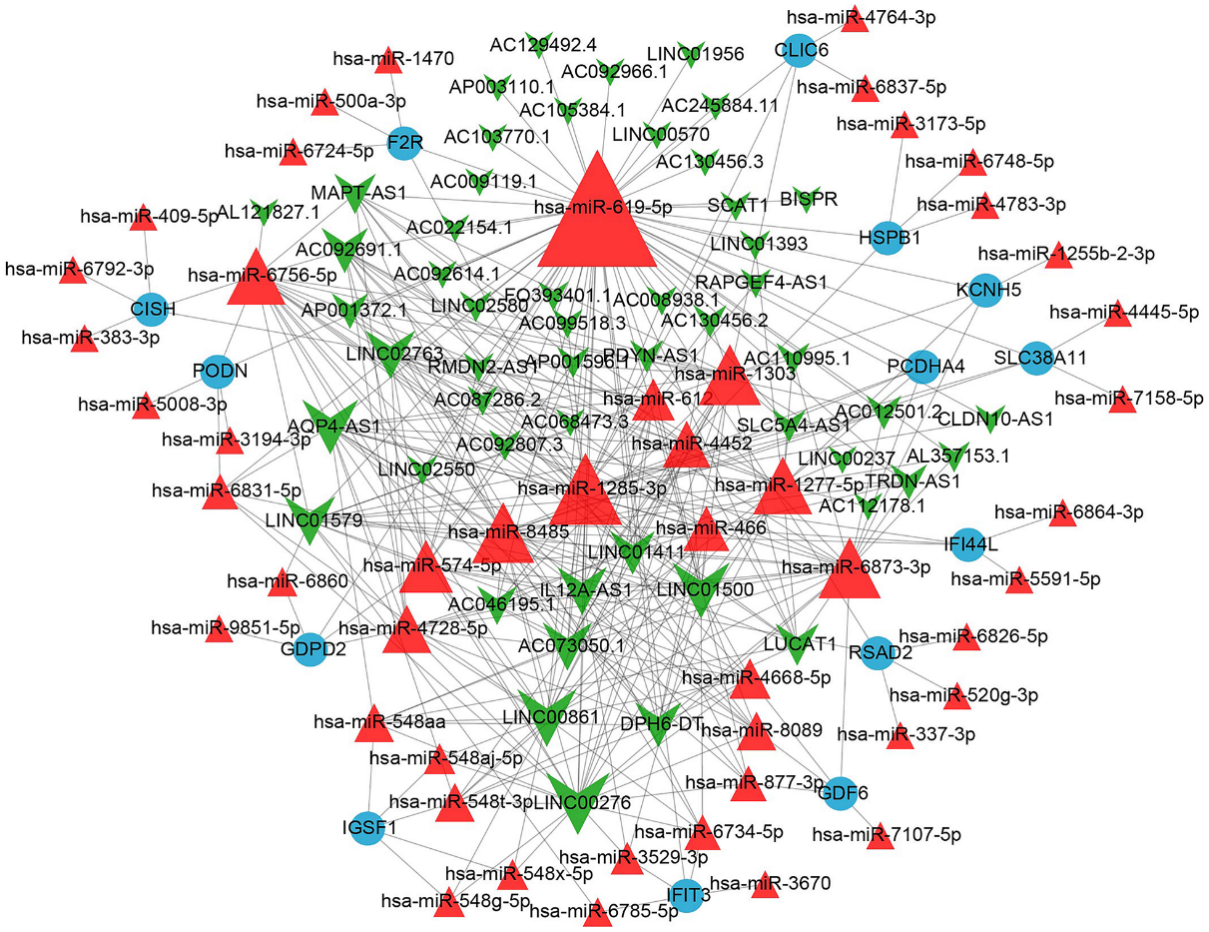
The miRNA-mRNA and miRNA-lncRNA network containing 52 lncRNA nodes, 49 miRNA nodes, 14 mRNA nodes and 351 edges.

A ceRNA network was constructed based on the relationships between the aforementioned 14 key mRNAs and miRNAs, as well as miRNAs and lncRNAs. The results are shown in Figure 7. This network contained 52 lncRNA nodes, 49 miRNA nodes, 14 mRNA nodes and 351 edges. The top 10 lncRNAs in the constructed ceRNA network are listed in Table 1.

**Table 1:** The top 10 lncRNAs in the constructed ceRNA network.

**Name**	**Degree**	**Regulation**	**Log_2_FC**	**P-value**
LINC00276	23	Down	-1.18932	0.005641
LINC00861	20	Up	1.05223	0.003525
LINC01500	19	Down	-0.65939	0.242896
AQP4-AS1	18	Up	0.934509	0.000185
LINC01579	15	Down	-1.11395	0.006746
AC073050.1	14	Up	0.643588	0.001204
IL12A-AS1	14	Up	0.68628	0.022851
LINC02763	14	Up	0.694538	0.049444
AC092691.1	12	Up	0.833615	0.000018
DPH6-DT	12	Down	-0.59975	0.01069956

lncRNA, long non-coding RNA; AS, antisense; ceRNA, competing endogenous RNA; FC, fold change.

### Identification of the potential lncRNA-miRNA-mRNA regulatory axis

From the above network, LINC00276 (degree = 23) and miR-619-5p (degree = 58) exhibited the highest degrees among lncRNAs and miRNAs, respectively. All 14 mRNA nodes had the same degree value. Interferon-induced protein with tetratricopeptide repeats 3 (IFIT3) has a binding site with miR-619-5p, according to a review of the literature [8]. The LINC00276- miR-619-5p-IFIT3 axis was identified.

### Senescent cell model construction and lncRNA expression verification

**Figure 8:**
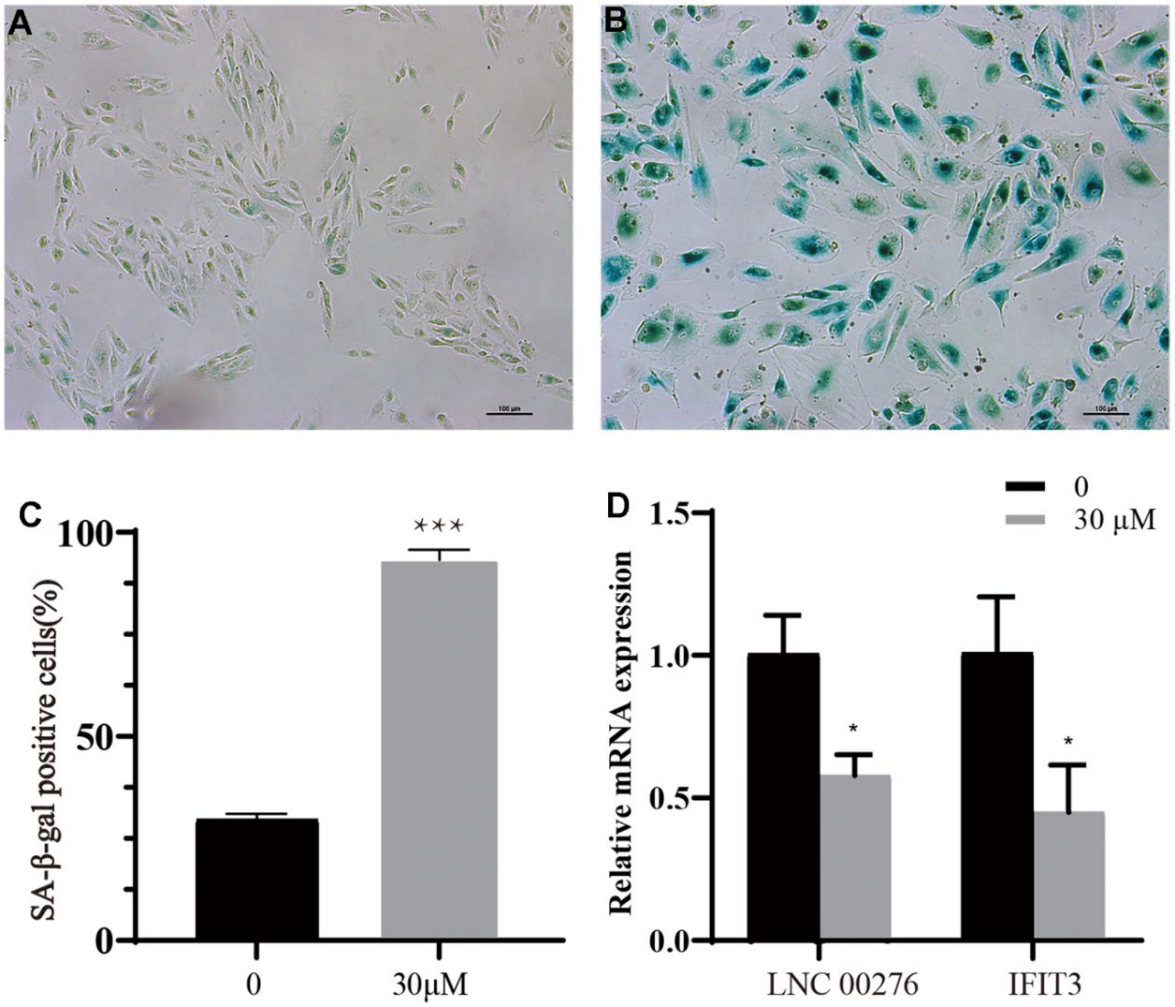
**Cell senescence analysis.** (**A**) Control group. (**B**) t-BH (30 μM) group. (**C**) Proportion of senescent cells in groups A and B. *** means P<0.001 compared with control group. After the senescence model was established, the cell body was enlarged and stained blue by the reagent (scale bar indicated as 100 μm). The percentage of senescent cells was significantly increased in group treated with t-BH compared with normal group through SA-β-gal staining compared with control group. (**D**) By using RT-PCR, the mRNA levels of and IFIT3 were detected. The expression of LINC00276 and IFIT3 was dramatically downregulated in the senescent cell as compared to the control group, * means P<0.05 compared with control group.

A senescent cell model was successfully constructed. The percentage of senescent cells was significantly increased in group treated with t-BH compared with normal group through SA-β-gal staining (Figure 8A–8C, *** means P<0.001 compared with control group). The expression of LINC00276 and IFIT3 was dramatically downregulated in the senescent cell as compared to the control group, which was consistent with the bioinformatic prediction outcomes (Figure 8D, * means P<0.05 compared with control group).

### LINC00276 can regulate cell migration

**Figure 9:**
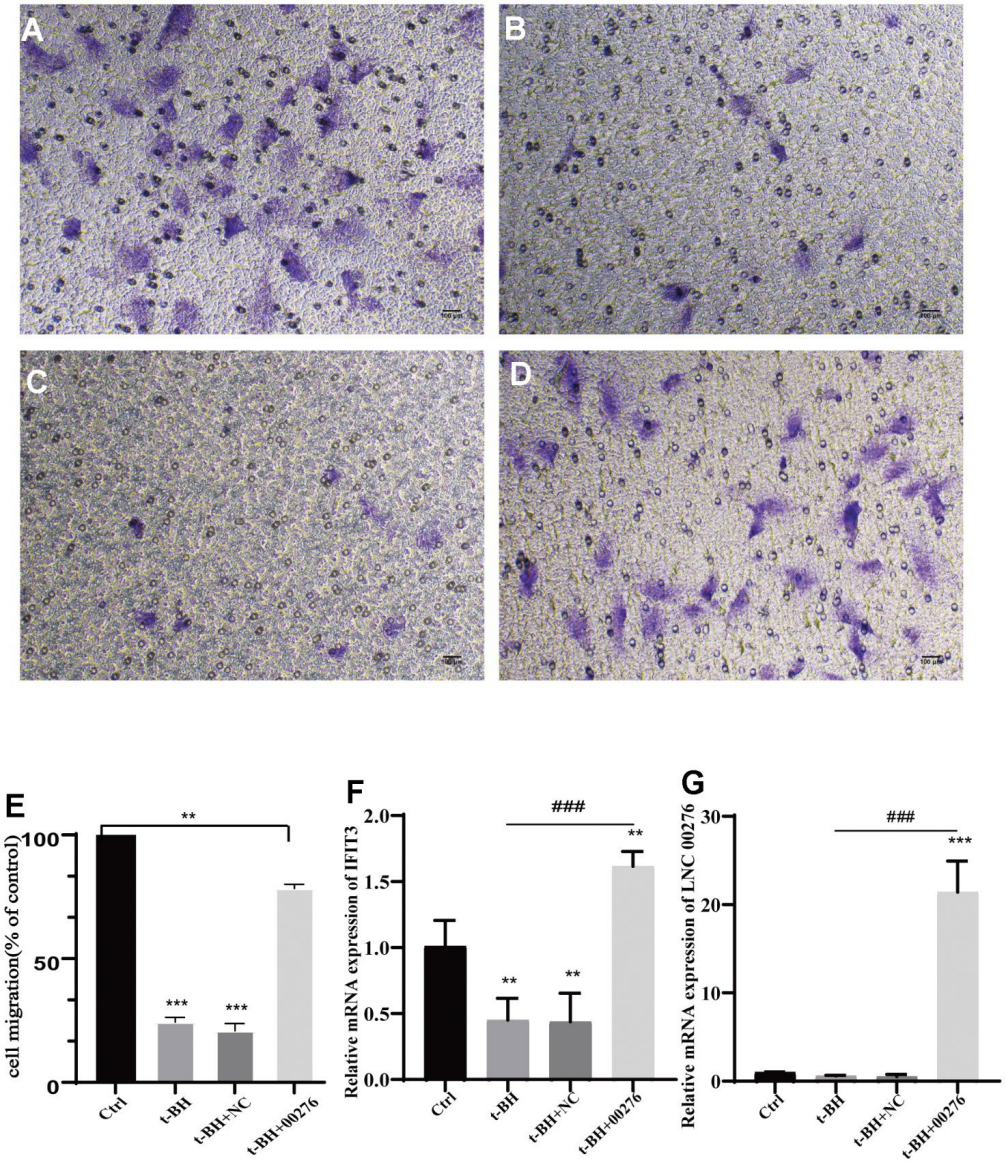
**Cell migration analysis.** (**A**) Control group. (**B**) t-BH group. (**C**, **D**) RPE-19 cells after t-BH treatment. (**C**) is the negative control. (**D**) is transfected with LINC00276 overexpression vector, cell migration rate was significantly reduced in the senescent cell model. (**E**) Migration rate of cells was determined using Transwell assays. (**F**, **G**) mRNA levels of IFIT3 and LINC00276 were detected by RT-PCR. The expression of LINC00276 was significantly improved after plasmid construction and stable transduction. After LINC00276 was upregulated, the migration rate was improved. *** means P<0.001, ** means P<0.01 compared with control group, ### means P<0.001 compared with t-BH group. NC means negative control.

After plasmid construction and stable transduction, the expression of LINC00276 was significantly improved (Figure 9G). The expression of IFIT3 was also upregulated (Figure 9F). We found that the cell migration rate was significantly reduced in the senescent cell model (Figure 9A, 9B); however, after LINC00276 was upregulated, the migration rate was improved (Figure 9C–9E).

Furthermore, fewer adherent APRE-19 cells were seen after t-BH treatment compared with the blank group in the Transwell assay. Moreover, the number of adherent cells increased after overexpressing LINC00276 in senescent cells. The results of Transwell assay confirmed that overexpression of LINC00276 could enhance the adhesion and penetration of senescent cells.

### LINC00276 can regulate wound healing in cells

**Figure 10:**
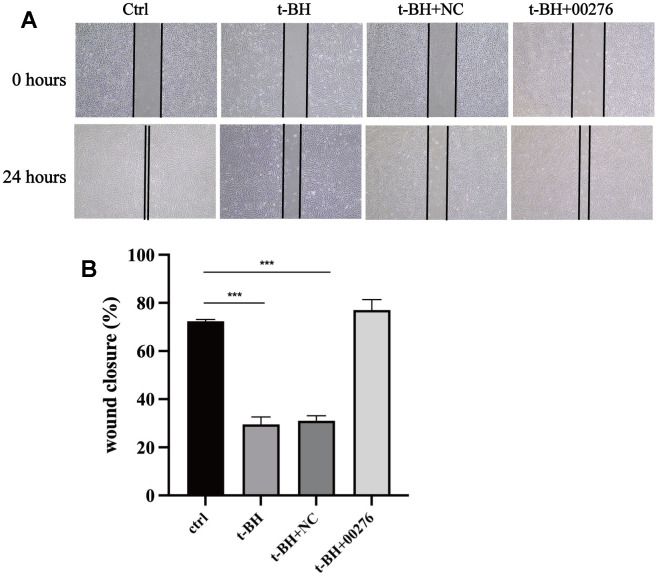
**Cells wound healing analysis.** (**A**) The wound area was marked with two black lines. The wound healing ability of APRE-19 cells after t-BH treatment was attenuated compared with the blank group. (**B**) Wound healing ability of the cells was significantly improved after LINC00276 overexpression. *** means P<0.001 compared with control group. NC means negative control.

In the cell scratch test, the wound healing ability of APRE-19 cells after t-BH treatment was attenuated compared with the blank group (Figure 10A), and the wound healing ability of the cells was significantly improved after LINC00276 overexpression (Figure 10B).

## DISCUSSION

A leading factor in global irreversible blindness is AMD [9]. Many factors may contribute to the disease. Inflammation plays prominent roles in AMD. In aging or diseases such as AMD, the balance between pro- and antioxidative signaling is often disrupted, which leads to related cytokine production and inflammatory responses [10, 11]. In this process, some proinflammatory cytokines are secreted [12].

LncRNAs are RNA molecules with lengths greater than 200 nucleotides. They have many regulatory functions in biochemical processes [11, 13]. They are also crucial for immune system control, have the potential to control the immune system, and cause the emergence of autoimmune disorders [14]. MiRNAs are noncoding RNAs 18 to 22 nucleotides in length [15], that have critical functions in many processes, such as metabolism, the immune cell response, inflammation, and especially neuroinflammatory activation and suppression [16, 17].

In our study, we found through bioinformatic analysis that the LINC00276-miR-619-5p-IFIT3 axis may play a role in AMD. *In vitro* experiments showed that LINC00276 and IFIT3 were downregulated in the t-BH-induced APRE-19 senescent cell model. Overexpression of LINC00276 could induce the migration of APRE-19 cells with IFIT3 upregulation.

LINC00276 was reported to be related to RPE cell differentiation. In APRE-19 cells incubated for 4 months, which exhibit naïve cell characteristics, LINC00276 was found to be highly expressed. LINC00276 can simultaneously regulate the expression of RPE-specific genes, this process may be mediated through interaction with heterogeneous nuclear ribonucleoproteins, (hnRNP) [18–20]. Because of the presence of two putative micro peptides encoded in LINC00276 that may play a function in the regulation of RPE differentiation [21]. Proinflammatory cytokines can induce loss of RPE characteristics with the altered expression of LINC00276. The NF-κB signaling pathway may regulate the expression of LINC00276 [22]. LINC00276 plays a crucial role in cellular regulation and protein binding, highlighting its importance as a potential prognostic marker for colorectal cancer (CRC) [23, 24]. In the context of breast cancer, LINC00276 may influence the activity of Inflammatory and Metabolic-Associated Adaptive Genes (IMAAGs), which are linked to the metastasis and recurrence of breast cancer, indicating the potential of LINC00276 in influencing breast cancer progression and recurrence [25].

IFIT3 has a binding site for miR-619-5p [8]. IFIT3 has a close relationship with neuroinflammation and the senescence process. In human brain vascular pericytes coculture with human cerebral microvascular endothelial cells models, the expression of interferon-associated proteins, including IFIT3, was upregulated approximately 2.5- to 8.5-log2-fold along with the function of restricting viruses [26]. In senescent biliary epithelial cells, Overexpression of IFIT3 was found, and it may be connected to the etiology of primary biliary cholangitis [27]. According to Wang et al. [28], it was hypothesized that IFIT3 may contribute to the cellular senescence process, a crucial step in the pathogenesis of AMD, through the overactive cyclic GMP-AMP synthase/stimulator of interferon genes (cGAS/STING) signaling pathway [29].

To further prove the above hypothesis, we used a t-BH-induced APRE-19 senescent cell model for experiments. SA-β-gal was detected to confirm the successful establishment of the senescent cell model. We found that LINC00276 and IFIT3 were significantly downregulated in senescent cells, and the cell migration rate was also significantly reduced. Studies have demonstrated that RPE cell migration is downregulated by RPE dysfunction, which may further contribute to the development of AMD [30, 31]. Our findings imply that LINC00276 might be crucial in the pathophysiology of AMD. We used lentivirus transduction to overexpress LINC00276 in senescent cells, and we found that the expression of IFIT3 was upregulated and that the cell migration rate was also increased. Taken together, these findings suggest that LINC00276 may be a possible therapeutic target for AMD.

Our research also has some limitations. First, numerous DEGs were left out of the analysis, and these DEGs might be essential in the pathogenesis of AMD. Second, we only performed a *silico*-based study, and detailed experiments are necessary to prove the relationship between the axis and AMD.

In conclusion, we established a lncRNA-miRNA-mRNA network through comprehensive bioinformatics analysis, providing a new perspective on the pathogenesis of AMD. *In vitro* experiment, LINC00276 and IFIT3 were downregulated in t-BH-induced APRE-19 senescent cell model. Overexpression of LINC00276 can induce the migration of APRE-19 cells with IFIT3 upregulation. LINC00276 may regulate the neuroinflammatory and cellular senescence processes through IFIT3. For this association to be validated, further in-depth studies are required.

## MATERIALS AND METHODS

### GEO dataset collection

From the GEO database, the RNA-seq data of RPE/retina samples from AMD patients and controls were retrieved (https://www.ncbi.nlm.nih.gov/geo/) (GSE135092) [32]. Eyes were obtained postmortem from 99 donors without ocular disease history and 23 donors with previously diagnosed AMD for this study. AMD was defined as any advanced AMD according to the Age-Related Eye Disease Study grading system [33]. Annotations were performed using the idmap1 R package to obtain the expression matrices for lncRNAs and mRNAs, information about the chromosomal locations, as well as the start and end positions of the genes.

### Analysis of DEGs

There were two sample sources in GSE135092: retinal tissue and RPE/choroid tissue from the AMD group and normal group, respectively, and each sample was divided into macular or nonmacular (peripheral) regions. We separated these samples into different groups and performed 6 comparisons as follows:

1. Retina of the macular region in AMD group vs. retina of the macular region in control group;
2. RPE of the macular region in AMD group vs. RPE of the macular region in control group;
3. Retina of the macular region in AMD group vs. retina of the nonmacular region in AMD group;
4. RPE of the macular region in AMD group vs. RPE of the nonmacular region in AMD group;
5. Retina of the macular region in control group vs. retina of the nonmacular region in control group;
6. RPE of the macular region in control group vs. RPE of the nonmacular in control group.

Among them, we focused on the difference analysis for groups (1) and (2). The other sets of differential genes were used to eliminate false positive results in groups (1) and (2). After principal component analysis (PCA) of the corresponding samples, R software’s edgeR [34, 35] module was used to find lncRNAs and mRNAs that expressed differently with P<0.05 and |log2-fold fold change (FC)| thresholds >0.585 as the cut-off criteria in the identification of DEGs. The volcano map and two-way hierarchical clustering analysis heat map were obtained to visualize the results.

### Functional enrichment analysis of the DEGs

Using DAVID [36], functional enrichment analysis of the differentially expressed mRNAs (DE-mRNAs) was carried out. We visualized the results of the enrichment analysis with bubble charts. To ensure the readability of the results, we selected the top 20 significantly enriched terms (P <0.05) for each item.

### PPI network of the DEGs

We predicted the PPI network of DE-mRNAs using the Search Tool for the Retrieval of Interacting Genes/Proteins (STRING) database [37] (STRING V11.0; http://string-db.org/). Then, we visualized the obtained PPI network using Cytoscape software (http://www.cytoscape.org, version 3.8.2; Institute for Systems Biology, Seattle, WA, USA), and the degree of each protein node was calculated.

### Coexpression analysis of lncRNAs and mRNAs

Pearson correlation analysis was performed on the differentially expressed lncRNAs and mRNAs from the previous analysis with |cor|> 0.6 and P <0.01 as the thresholds for significance.

### GO and KEGG functional enrichment analysis

The analysis was carried out based on the coexpression results. We used the clusterProfiler package [38] to assess functional enrichment (GO biological processes [BPs]) and pathway enrichment (KEGG) in the R language, and the screening threshold for significantly enriched functions/pathways was P <0.05.

### Extraction of gene sequences

Based on the above results, we obtained all the significantly coexpressed lncRNAs and mRNAs from all the DEGs. We downloaded the cDNA sequences of the lncRNAs and mRNAs (according to the chromosome on which the gene was located and the start and end positions of the gene annotation information) from REST API endpoints (https://rest.ensembl.org/) provided by the Ensemble database for further analysis. All the sequences of human mature miRNAs were also downloaded from miRbase.

### External data validation

The research received approval from the relevant Institutional Review Board. We obtained the DEG results from another AMD-related dataset, GSE115828 [39, 40], from the GEO; genes with P < 0.05 in this dataset were overlapped with the above results. The obtained mRNAs were used for subsequent functional analysis using Metascape [41].

### Construction of the lncRNA-miRNA-mRNA competing endogenous RNA (ceRNA) networks

We used miRanda software to perform combined analysis on lncRNA-miRNA and mRNA-miRNA interactions. The analysis parameters are Score Threshold: 160, Energy Threshold: -10.000000 kcal/mol; other analysis parameters are the software defaults.

We used the miRNA with a score of the top 5 in the miRanda analysis as the most likely result of regulating the corresponding mRNA in this analysis. Then, according to the selected miRNAs, we selected the relationship pair of total Energy <-200 as a reliable result to obtain the corresponding regulatory relationship of lncRNA-miRNA from the miRanda analysis results.

Based on the corresponding regulatory relationships between lncRNA-miRNA and mRNA-miRNA obtained from the above analysis, Cytoscape software was used to construct and visualize a ceRNA regulatory relationship network (Figure 7).

### Identification of the potential lncRNA-miRNA-mRNA regulatory axis

LncRNAs and miRNAs with the highest degrees were extracted. Furthermore, through a literature review, the related mRNAs were identified. In this way, the potential lncRNA-miRNA-mRNA regulatory axis was determined.

### Cell culture and cell model construction

ARPE-19 cells were purchased from the American Type Culture Collection (ATCC, Manassas, MD, USA) at passage 10 and was cultured in DMEM/F12 (12400-024, Gibco, USA) medium containing 10% FBS (10099-141, Gibco). Senescence was induced in ARPE-19 cells by using tert-butyl hydroperoxide (t-BH, 458139, Sigma-Aldrich, USA). A medium containing 30 μM t-BH was substituted for the original media after the cells had been cultured for 24 hours, and the cells were placed in a 37° C CO_2_ incubator for 2 hours, and then replaced with DMEM/F12 medium containing 10% FBS, the procedure of t-BH treatment was repeated five times and lasted for 5 days, then the cells were recovered for 3 days.

### Cell senescence analysis

Senescence Cells Histochemical Staining Kits were used to identify the senescent cells (CS0030, Sigma). Cell models were created after 24 hours of cell culture in a 24-well plate, after which the cells were washed with PBS, treated in Fixation Buffer for 7 minutes under the room temperature circumstance, and then the cells were washed once more. Cells were then incubated in accordance with the kit’s directions. The cells were captured by Nikon Ts2FL microscope (×200), and both the total number of cells and blue-stained cells were recorded.

### Lentiviral transfer vector construction and stable transduction

It was decided to use the lentiviral transfer vector (pEZ-Lv201). Following a standardized procedure, highly purified plasmids, EndoFectin-Lenti™ and TiterBoost™ reagents were used to create the lentiviral particles. Lenti-Pac™ HIV packaging mix and the lentiviral transfer vector were co-transfected into 293Ta cells (Cat #: LT008). The lentivirus particles were cleaned and kept in aliquots at -80° C (purified particles). After co-infecting t-BH-pretreated cells with the lentiviral vector LINC00276 (GeneCopoeia, China), a stable cell line was discovered through puromycin screening. Negative control cell lines were generated via infection with control lentivirus containing a random sequence (control vectors).

### RNA extraction and RT-PCR

According to the manufacturer’s guidelines, total cellular RNAs were isolated from RPE cells using the SteadyPure Universal RNA Extraction Kit from Accurate Biotechnology (Hunan) Co., Ltd., China. M-MLV Reverse Transcriptase (Accurate Biotechnology (Hunan) Co., Ltd., China) was used to create cDNA. Using PCR SYBR Green buffer, mRNA and lncRNA were analyzed by RT-PCR (Takara Bio Inc., Japan). The ABI StepOnePlus™ Real-Time PCR System performed 40 cycles of PCR for 5 seconds at 95° C and for 30 seconds at 60° C (Applied Biosystems Inc., Waltham, MA, USA). With GAPDH serving as an internal control, the 2[-Delta C(T)] technique was used to calculate relative expression. Table 2 contains a list of the primers used.

**Table 2:** Primers of LINC00276 and IFIT3.

**Primer**	**5**’**-3**’ **Sequence**	
	**Forward**	**Reverse**
LINC00276	GAGGTCCTGAAGAAAAGGGAAAAC	TCCTTATCCAGTCCACCATTGA
IFIT3	GAACATGCTGACCAAGCAGA	CAGTTGTGTCCACCCTTCCT
GAPDH	CAAGAGCACAAGAGGAAGAGAG	CTACATGGCAACTGTGAGGAG

### Scratch assay

After the cells were cultured in a 6-well plate for 24 hours, they were “wounded” with a pipette tip. The cells were then washed with PBS (8121541, Gibco), replaced with serum-free DMEM/F12 medium. Per wound, two photos were taken at a defined position (Nikon Ts2FL microscope) after scratch (0h) and 24h (×40). The ImageJ (program Fiji (ImageJ-win64; https://imagej.net/Fiji)) was used to measure the wound area and the wound healing rate was calculated.

### Cell migration

The cells were collected, and 200 μL of the cells diluted to 1.0×10^5^ cells/mL with serum-free DMEM/F12 medium was inoculated into the upper chamber of Transwell (pore size: 8 μm). The DMEM/F12 medium containing 10% FBS was then added to the lower chamber. The chamber was placed in a 37° C CO_2_ incubator for 24 hours. Then the cells were fixed with 4% paraformaldehyde for 20 minutes, and stained with 0.1% crystal violet (G1063, Solarbio, China) in the dark for 20 minutes. The cells left in the upper chamber were wiped away with a cotton swab. The migrated cells were photographed using Nikon Ts2FL microscope (×200).

### Statistical analysis

At least three separate independent runs of each experiment were completed. Software from GraphPad Prism 8.0.2 and SPSS (SPSS lnc., Chicago, IL, USA) were used for all statistical analyses (GraphPad, La Jolla, CA, USA). The mean and SD (standard deviation) from triplicates are used to represent all data. Independent sample t-tests were used to assess differences between two groups, and one-way ANOVA was used to analyze differences between several groups. Statistics determined that differences with a P-value <0.05 were statistically significant.
